# 2,2-Diphenyl­benzo[*c*]quinoline-1-ox­yl

**DOI:** 10.1107/S1600536809013476

**Published:** 2009-04-18

**Authors:** Corrado Rizzoli, Elda Marku, Lucedio Greci, Paola Astolfi

**Affiliations:** a Dipartimento di Chimica Generale ed Inorganica, Chimica Analitica, Chimica Fisica, Universitá degli Studi di Parma, Viale G. P. Usberti 17/A, I-43100 Parma, Italy; b Fakulteti i Shkencave të Natyrës, Departamenti i Kimise, Universiteti i Tiranes, Bulevardi "Zogu I", Tirana, Albania; c Dipartimento ISAC, Universitá Politecnica delle Marche, Via Brecce Bianche, I-60131 Ancona, Italy

## Abstract

In the title compound, C_25_H_18_NO, a stable phenanthridinic nitroxide, the ring containing the nitroxide function assumes a twist-boat conformation and the dihedral angle formed by adjacent benzene rings is 21.78 (5)°. The phenyl substituents at position 2 are approximately orthogonal to each other, forming a dihedral angle of 81.04 (4)°. The crystal structure is stabilized by an intra­molecular C—H⋯O hydrogen bond and by C—H⋯π inter­actions.

## Related literature

For applications of nitroxides in biology, see: Carloni *et al.* (1996 ▶); Greci (1982 ▶); Likhtenshtein *et al.* (2008 ▶). For their applications in medicine, see: Damiani *et al.* (2008 ▶); Krishna *et al.* (1996 ▶). For their use in pharmacology and cosmetics, see: Krishna *et al.* (1996 ▶); Setjurc *et al.* (1995 ▶); Greci *et al.* (2007 ▶). For their applications in chemical processes and materials science, see: Guillaneuf *et al.* (2007 ▶); Arends *et al.* (2006 ▶); Franchi *et al.* (2008 ▶); Bailly *et al.* (2006 ▶); Bugnon *et al.* (2007 ▶). For a description of the Cambridge structural Database, see: Allen (2002 ▶); For puckering parameters, see: Cremer & Pople (1975 ▶). For graph-set motifs, see: Etter *et al.* (1990 ▶). For the synthesis, see: Colonna *et al.* (1980 ▶).
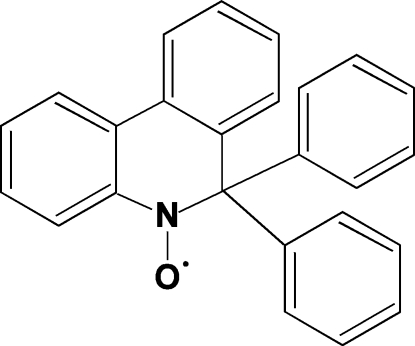


## Experimental

### 

#### Crystal data

C_25_H_18_NO
*M* *_r_* = 348.40
Monoclinic, 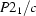

*a* = 12.6188 (12) Å
*b* = 8.8704 (8) Å
*c* = 16.6083 (15) Å
β = 102.998 (2)°
*V* = 1811.4 (3) Å^3^
*Z* = 4
Mo *K*α radiation
μ = 0.08 mm^−1^
*T* = 295 K
0.16 × 0.14 × 0.08 mm

#### Data collection

Bruker SMART 1000 CCD diffractometer
Absorption correction: multi-scan (*SADABS*; Bruker, 1998 ▶) *T*_min_ = 0.972, *T*_max_ = 0.990
18472 measured reflections
3548 independent reflections
2115 reflections with *I* > 2σ(*I*)
*R*_int_ = 0.047

#### Refinement

*R*[*F* ^2^ > 2σ(*F* ^2^)] = 0.037
*wR*(*F* ^2^) = 0.080
*S* = 1.01
3548 reflections
244 parameters
H-atom parameters constrained
Δρ_max_ = 0.11 e Å^−3^
Δρ_min_ = −0.14 e Å^−3^

### 

Data collection: *SMART* (Bruker, 1998 ▶); cell refinement: *SAINT-Plus* (Bruker, 1998 ▶); data reduction: *SAINT-Plus*; program(s) used to solve structure: *SIR97* (Altomare *et al.*, 1999 ▶); program(s) used to refine structure: *SHELXL97* (Sheldrick, 2008 ▶); molecular graphics: *ORTEP-3 for Windows* (Farrugia, 1997 ▶) and *SCHAKAL97* (Keller, 1997 ▶); software used to prepare material for publication: *SHELXL97*, *PARST95* (Nardelli, 1995 ▶) and *WinSim* (Duling, 1994 ▶).

## Supplementary Material

Crystal structure: contains datablocks global, I. DOI: 10.1107/S1600536809013476/fb2149sup1.cif

Structure factors: contains datablocks I. DOI: 10.1107/S1600536809013476/fb2149Isup2.hkl

Additional supplementary materials:  crystallographic information; 3D view; checkCIF report

## Figures and Tables

**Table 1:** Hydrogen-bond geometry (Å, °)

*D*—H⋯*A*	*D*—H	H⋯*A*	*D*⋯*A*	*D*—H⋯*A*
C21—H21⋯O1	0.93	2.42	3.044 (2)	124
C24—H24⋯*Cg*1^i^	0.93	3.27	3.864 (3)	136
C6—H6⋯*Cg*2^ii^	0.93	3.16	3.932 (4)	142
C10—H10⋯*Cg*3^iii^	0.93	2.97	3.839 (4)	154

Symmetry codes: (i) 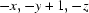; (ii) 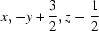; (iii) 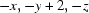. *Cg*1, *Cg*2 and *Cg*3 are the centroids of the C2–C7, C14–C19 and C20–C25 aromatic rings, respectively.

**Fig. 1.**
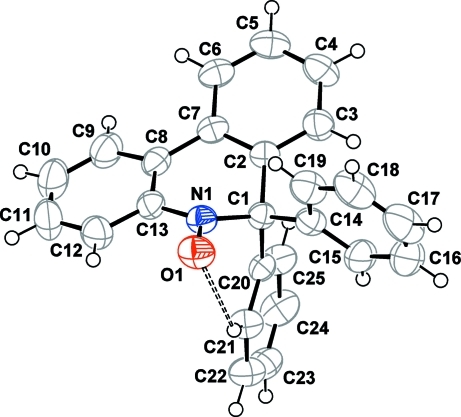
The molecular structure of the title compound. The displacement ellipsoids are drawn at the 50% probability level. The intramolecular C—H···O hydrogen bond is shown as a dashed line.

**Fig. 2.**
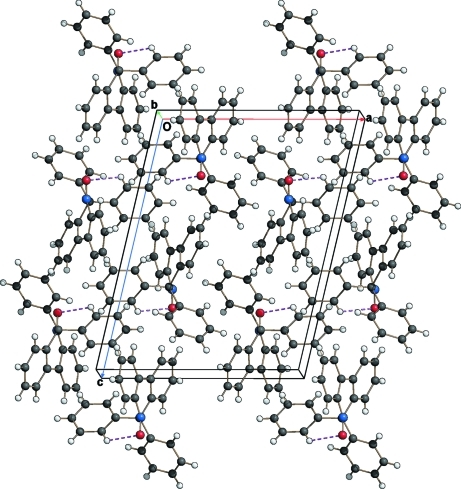
Crystal packing of the title compound viewed approximately along the b axis. Intramolecular C—H···O hydrogen bonds are shown as dashed lines.

**Fig. 3.**
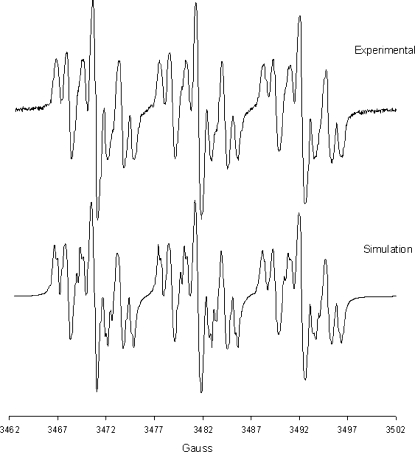
Experimental and simulated ESR spectrum of the title compound.

## supplementary crystallographic information

### Comment

Most nitroxides (aminoxyls) are stable radicals that have received a great attention since the second half of the last century for the variety of their applications. In fact, in biology they have been used as relatively stable spin-adducts for studying short-lived radicals such as superoxide (Carloni *et al.*, 1996), hydroxy and alkylperoxy radicals (Greci, 1982) that are typical for peroxidation processes. In this field, nitroxides have also been used as spin probes/spin labels for studying membranes and proteins (Likhtenshtein *et al.*, 2008). In medicine, they have been studied as mimics of superoxide dismutase (Damiani *et al.*, 2008), catalase (Krishna *et al.*, 1996) and as contrast agents of NMR-imaging. In pharmacology, they have been used to study the metabolism of drugs (Setjurc *et al.*, 1995). As antioxidants they have been used in polymers, in stabilising monomers for polyaddition during controlled radical polymerization, for the synthesis of living polymers and in large hydrocarbon distilleries for preventing polymerization and incrustation of pipes (Guillaneuf *et al.*, 2007). As antioxidants, they have also been studied in the medical (ischemia-reperfusion) and cosmetic field for protecting against free radical damage (Greci *et al.*, 2007). In chemistry, they have been used as inhibitors of radical processes, in radical synthesis and as mediators of controlled oxidations of primary alcohols and aldehydes (Arends *et al.*, 2006). Recently, nitroxides have found applications in supramolecular chemistry (Franchi *et al.*, 2008), in nanomaterials (Bailly *et al.*, 2006) and in other technologies such as the construction of free radical batteries (Bugnon *et al.*, 2007). In view of its potential application in cosmetics and as a precursor of alkoxyamines used in the controlled radical polymerization, the title compound has been synthesized and its crystal structure is reported here.

In the molecule of the title compound (Fig. 1), geometric parameters are usual. The value of the N1-O1 bond length (1.2811 (13) Å) corresponds well to the mean value of 1.286 (1) Å found from 891 observations yielded by the Cambridge Crystallographic Database (version 5.30; Allen, 2002) for the N—O single bond, and is in agreement with the radical character of the oxygen atom evidenced by an ESR study (Fig. 3). The benzoquinoline ring system is not planar, the dihedral angle between the C2–C7 and C8–C13 benzene rings being 21.78 (5)° as a result of the *sp*^3^ character of the C1 carbon atom. The ring containing the nitroxide function assumes a twist-boat conformation, with puckering parameters *Q* = 0.443 (2) Å, θ = 110.23 (18) ° and φ = -142.62 (18) ° (Cremer & Pople, 1975). The dihedral angle formed by the phenyl substituents at C1 is 81.04 (4)°. The molecular conformation is stabilized by an intramolecular C—H···O hydrogen bond (Tab. 1) generating an *S*(5) ring motif (Etter *et al.*, 1990). In the crystal packing (Fig. 2), weak C—H···π interactions are observed ranging from 2.97 to 3.27 Å (Table 1).

### Experimental

A dried tetrahydrofuran solution (30 ml) of 2-phenyl-3,4-benzoquinoline-N-oxide (2.72 g, 10 mmol) prepared according to the literature method (Colonna *et al.*,1980), was reacted at room temperature with phenylmagnesium bromide (3.62 g, 20 mmol; a commercial compound produced by Aldrich). The reaction mixture was then poured into a 10% ammonium chloride water solution (100 ml) and extracted with diethyl ether. The dried organic layer was oxidised with lead dioxide (4.8 g, 20 mmol) and, after filtration, evaporated to dryness. The residue  was chromatographed on silica gel column eluting with cyclohexane/ethyl acetate (8:2 v/v; 300 mL). The expected nitroxide was isolated from the red fraction in 83% yield (2.9 g): m.p 176-7°C (175°C in Colonna *et al.*, 1980). Single crystals suitable for X-ray analysis were obtained by slow evaporation of an ethanol solution. IR in KBr, ν, cm^-1^: 1926, 1771, 1732, 1667, 1589, 1732. Mass, calcd. for C_25_H_18_NO, 348.44; found: *m/z* = 349(*M*^+^+1, 22.8), 348(60.6), 318(87.1), 272(47.4), 254(66.8), 240 (100). EPR, hfccs in Gauss: a_N_ = 10.75; a_H_ = 2.76; a_H_ = 2.67; a_H_ = 1.03; a_H_ = 0.88; a_H_ = 0.38; a_H_ = 0.27; g-value = 2.0057_7_. The melting point was measured on a Mitamura Riken Kogyo Mp. D electrochemical apparatus and was not corrected. IR spectrum were recorded in KBr with a Perkin–Elmer MGX1 spectrophotometer equipped with Spectra Tech. Mass spectrum was recorded on a Carlo Erba QMD 1000 mass spectrometer in positive electron impact (EI) mode. The electron-spin resonance (ESR) spectrum (Fig. 3) was simulated by using WinSim program in the NIEHS Public ESR Software Tools package (Duling, 1994).

### Refinement

Though all the H atoms were discernible in the difference electron density maps, the H atoms were positioned into idealized positions with C—H = 0.93 Å, and refined using a riding model approximation with *U*_iso_(H) = 1.2 *U*_eq_(C).

### Crystal data

**Table:**

C_25_H_18_NO	*F*(000) = 732
*M**_r_* = 348.40	*D*_x_ = 1.278 Mg m^−^^3^
Monoclinic, *P*2_1_/*c*	Melting point = 449–450 K
Hall symbol: -P 2ybc	Mo *K*α radiation, λ = 0.71073 Å
*a* = 12.6188 (12) Å	Cell parameters from 1226 reflections
*b* = 8.8704 (8) Å	θ = 3.2–24.8°
*c* = 16.6083 (15) Å	µ = 0.08 mm^−^^1^
β = 102.998 (2)°	*T* = 295 K
*V* = 1811.4 (3) Å^3^	Block, red
*Z* = 4	0.16 × 0.14 × 0.08 mm

### Data collection

**Table:**

Bruker SMART 1000 CCD diffractometer	3548 independent reflections
Radiation source: fine-focus sealed tube	2115 reflections with *I* > 2σ(*I*)
graphite	*R*_int_ = 0.047
ω scans	θ_max_ = 26.0°, θ_min_ = 1.7°
Absorption correction: multi-scan (*SADABS*; Bruker, 1998)	*h* = −15→15
*T*_min_ = 0.972, *T*_max_ = 0.990	*k* = −10→10
18472 measured reflections	*l* = −20→20

### Refinement

**Table:**

Refinement on *F*^2^	Primary atom site location: structure-invariant direct methods
Least-squares matrix: full	Secondary atom site location: difference Fourier map
*R*[*F*^2^ > 2σ(*F*^2^)] = 0.037	Hydrogen site location: difference Fourier map
*wR*(*F*^2^) = 0.080	H-atom parameters constrained
*S* = 1.01	*w* = 1/[σ^2^(*F*_o_^2^) + (0.0321*P*)^2^] where *P* = (*F*_o_^2^ + 2*F*_c_^2^)/3
3548 reflections	(Δ/σ)_max_ < 0.001
244 parameters	Δρ_max_ = 0.11 e Å^−^^3^
0 restraints	Δρ_min_ = −0.14 e Å^−^^3^
72 constraints	

### Special details

**Table:**

Refinement. Refinement of *F*^2^ against ALL reflections. The weighted *R*-factor *wR* and goodness of fit *S* are based on *F*^2^, conventional *R*-factors *R* are based on *F*, with *F* set to zero for negative *F*^2^. The threshold expression of *F*^2^ > σ(*F*^2^) is used only for calculating *R*-factors(gt) *etc*. and is not relevant to the choice of reflections for refinement. *R*-factors based on *F*^2^ are statistically about twice as large as those based on *F*, and *R*- factors based on ALL data will be even larger.

### Fractional atomic coordinates and isotropic or equivalent isotropic displacement parameters (Å^2^)

**Table:**

	*x*	*y*	*z*	*U*_iso_*/*U*_eq_	
N1	0.26974 (9)	0.84150 (12)	0.17884 (7)	0.0436 (3)	
O1	0.29038 (8)	0.90766 (11)	0.24920 (6)	0.0590 (3)	
C2	0.29264 (10)	0.61901 (15)	0.09755 (8)	0.0406 (3)	
C20	0.13820 (10)	0.64080 (15)	0.17300 (7)	0.0386 (3)	
C13	0.22201 (11)	0.92305 (16)	0.10729 (9)	0.0443 (3)	
C8	0.21679 (11)	0.85600 (16)	0.03064 (8)	0.0458 (4)	
C14	0.33392 (11)	0.60524 (16)	0.25222 (8)	0.0445 (4)	
C1	0.25883 (11)	0.67236 (14)	0.17575 (8)	0.0391 (3)	
C7	0.26972 (11)	0.70981 (15)	0.02684 (8)	0.0424 (3)	
C6	0.30162 (12)	0.66030 (18)	−0.04392 (9)	0.0526 (4)	
H6	0.2896	0.7217	−0.0905	0.063*	
C25	0.07658 (13)	0.54387 (17)	0.11658 (9)	0.0550 (4)	
H25	0.1075	0.4964	0.0775	0.066*	
C3	0.34171 (12)	0.48029 (16)	0.09411 (9)	0.0537 (4)	
H3	0.3560	0.4186	0.1406	0.064*	
C21	0.08830 (12)	0.71122 (17)	0.22877 (9)	0.0512 (4)	
H21	0.1280	0.7785	0.2668	0.061*	
C22	−0.01884 (13)	0.68398 (18)	0.22926 (10)	0.0595 (4)	
H22	−0.0506	0.7328	0.2675	0.071*	
C5	0.35039 (12)	0.52270 (19)	−0.04600 (9)	0.0596 (4)	
H5	0.3703	0.4908	−0.0938	0.072*	
C23	−0.07899 (13)	0.58527 (19)	0.17372 (10)	0.0610 (4)	
H23	−0.1512	0.5656	0.1744	0.073*	
C12	0.18124 (13)	1.06672 (18)	0.11352 (10)	0.0621 (4)	
H12	0.1884	1.1122	0.1649	0.074*	
C15	0.30015 (13)	0.48928 (18)	0.29547 (9)	0.0603 (4)	
H15	0.2285	0.4560	0.2807	0.072*	
C24	−0.03136 (13)	0.51648 (19)	0.11758 (10)	0.0647 (5)	
H24	−0.0718	0.4501	0.0793	0.078*	
C9	0.16276 (13)	0.9348 (2)	−0.03924 (10)	0.0646 (5)	
H9	0.1562	0.8914	−0.0911	0.078*	
C19	0.44123 (12)	0.65176 (19)	0.27533 (9)	0.0614 (4)	
H19	0.4658	0.7291	0.2464	0.074*	
C4	0.36960 (13)	0.43243 (19)	0.02258 (10)	0.0622 (4)	
H4	0.4016	0.3384	0.0210	0.075*	
C10	0.11911 (15)	1.0748 (2)	−0.03309 (13)	0.0808 (6)	
H10	0.0821	1.1245	−0.0804	0.097*	
C11	0.13017 (15)	1.1411 (2)	0.04279 (13)	0.0782 (5)	
H11	0.1028	1.2375	0.0465	0.094*	
C18	0.51180 (15)	0.5847 (2)	0.34067 (11)	0.0777 (6)	
H18	0.5835	0.6178	0.3558	0.093*	
C16	0.37243 (17)	0.4214 (2)	0.36115 (10)	0.0838 (6)	
H16	0.3491	0.3426	0.3899	0.101*	
C17	0.47781 (18)	0.4703 (3)	0.38350 (11)	0.0866 (6)	
H17	0.5260	0.4257	0.4277	0.104*	

### Atomic displacement parameters (Å^2^)

**Table:**

	*U*^11^	*U*^22^	*U*^33^	*U*^12^	*U*^13^	*U*^23^
N1	0.0451 (7)	0.0437 (7)	0.0430 (7)	−0.0050 (5)	0.0120 (5)	−0.0072 (6)
O1	0.0672 (7)	0.0597 (7)	0.0499 (6)	−0.0065 (5)	0.0130 (5)	−0.0191 (5)
C2	0.0382 (8)	0.0459 (9)	0.0375 (8)	−0.0026 (7)	0.0083 (6)	−0.0042 (7)
C20	0.0412 (8)	0.0409 (8)	0.0328 (7)	−0.0012 (6)	0.0067 (6)	0.0045 (6)
C13	0.0407 (8)	0.0414 (9)	0.0518 (9)	−0.0035 (7)	0.0126 (7)	0.0040 (7)
C8	0.0435 (9)	0.0484 (9)	0.0465 (9)	−0.0048 (7)	0.0121 (7)	0.0064 (7)
C14	0.0442 (9)	0.0538 (9)	0.0347 (7)	0.0060 (7)	0.0073 (6)	−0.0024 (7)
C1	0.0428 (8)	0.0391 (8)	0.0350 (7)	−0.0008 (6)	0.0081 (6)	−0.0008 (6)
C7	0.0383 (8)	0.0501 (9)	0.0386 (8)	−0.0081 (7)	0.0085 (6)	−0.0028 (7)
C6	0.0482 (9)	0.0708 (11)	0.0396 (9)	−0.0065 (8)	0.0115 (7)	−0.0004 (8)
C25	0.0578 (10)	0.0598 (10)	0.0492 (9)	−0.0158 (8)	0.0159 (8)	−0.0097 (8)
C3	0.0627 (10)	0.0526 (10)	0.0460 (9)	0.0061 (8)	0.0128 (8)	−0.0018 (7)
C21	0.0474 (9)	0.0599 (10)	0.0467 (9)	−0.0056 (7)	0.0113 (7)	−0.0060 (7)
C22	0.0504 (10)	0.0726 (11)	0.0592 (10)	−0.0016 (8)	0.0205 (8)	−0.0002 (9)
C5	0.0520 (10)	0.0823 (13)	0.0468 (9)	−0.0012 (9)	0.0159 (8)	−0.0138 (9)
C23	0.0452 (9)	0.0765 (12)	0.0610 (10)	−0.0096 (9)	0.0112 (8)	0.0115 (9)
C12	0.0625 (11)	0.0494 (10)	0.0782 (12)	−0.0006 (8)	0.0241 (9)	−0.0012 (9)
C15	0.0582 (10)	0.0713 (11)	0.0513 (9)	0.0106 (8)	0.0119 (8)	0.0165 (8)
C24	0.0612 (11)	0.0725 (12)	0.0577 (10)	−0.0262 (9)	0.0076 (9)	−0.0069 (9)
C9	0.0687 (11)	0.0689 (12)	0.0558 (10)	0.0025 (9)	0.0129 (9)	0.0158 (9)
C19	0.0491 (10)	0.0752 (12)	0.0560 (10)	0.0062 (8)	0.0035 (8)	−0.0030 (9)
C4	0.0652 (11)	0.0634 (11)	0.0591 (10)	0.0113 (8)	0.0164 (8)	−0.0149 (9)
C10	0.0855 (14)	0.0726 (13)	0.0831 (14)	0.0150 (11)	0.0166 (11)	0.0324 (11)
C11	0.0866 (14)	0.0491 (11)	0.0998 (15)	0.0135 (9)	0.0229 (12)	0.0181 (11)
C18	0.0564 (11)	0.0990 (15)	0.0687 (12)	0.0196 (11)	−0.0048 (10)	−0.0123 (12)
C16	0.0889 (16)	0.0997 (15)	0.0638 (12)	0.0323 (12)	0.0192 (11)	0.0338 (11)
C17	0.0782 (15)	0.1184 (18)	0.0552 (11)	0.0486 (13)	−0.0020 (11)	0.0070 (12)

### Geometric parameters (Å, °)

**Table:**

N1—O1	1.2811 (13)	C22—C23	1.371 (2)
N1—C13	1.4057 (17)	C22—H22	0.9300
N1—C1	1.5064 (16)	C5—C4	1.369 (2)
C2—C3	1.3844 (18)	C5—H5	0.9300
C2—C7	1.3994 (18)	C23—C24	1.362 (2)
C2—C1	1.5305 (17)	C23—H23	0.9300
C20—C25	1.3763 (18)	C12—C11	1.375 (2)
C20—C21	1.3810 (18)	C12—H12	0.9300
C20—C1	1.5383 (18)	C15—C16	1.392 (2)
C13—C12	1.387 (2)	C15—H15	0.9300
C13—C8	1.3933 (18)	C24—H24	0.9300
C8—C9	1.3942 (19)	C9—C10	1.372 (2)
C8—C7	1.4665 (19)	C9—H9	0.9300
C14—C15	1.3760 (19)	C19—C18	1.375 (2)
C14—C19	1.385 (2)	C19—H19	0.9300
C14—C1	1.5244 (17)	C4—H4	0.9300
C7—C6	1.3961 (19)	C10—C11	1.369 (2)
C6—C5	1.371 (2)	C10—H10	0.9300
C6—H6	0.9300	C11—H11	0.9300
C25—C24	1.387 (2)	C18—C17	1.363 (3)
C25—H25	0.9300	C18—H18	0.9300
C3—C4	1.3796 (19)	C16—C17	1.368 (3)
C3—H3	0.9300	C16—H16	0.9300
C21—C22	1.375 (2)	C17—H17	0.9300
C21—H21	0.9300		
			
O1—N1—C13	119.72 (11)	C21—C22—H22	119.8
O1—N1—C1	119.05 (10)	C4—C5—C6	119.76 (14)
C13—N1—C1	117.73 (11)	C4—C5—H5	120.1
C3—C2—C7	119.14 (13)	C6—C5—H5	120.1
C3—C2—C1	121.50 (12)	C24—C23—C22	118.95 (15)
C7—C2—C1	119.32 (12)	C24—C23—H23	120.5
C25—C20—C21	117.79 (13)	C22—C23—H23	120.5
C25—C20—C1	122.53 (12)	C11—C12—C13	119.18 (16)
C21—C20—C1	119.68 (12)	C11—C12—H12	120.4
C12—C13—C8	121.22 (14)	C13—C12—H12	120.4
C12—C13—N1	120.37 (13)	C14—C15—C16	120.49 (16)
C8—C13—N1	118.42 (13)	C14—C15—H15	119.8
C13—C8—C9	117.42 (14)	C16—C15—H15	119.8
C13—C8—C7	119.23 (12)	C23—C24—C25	121.02 (15)
C9—C8—C7	123.33 (14)	C23—C24—H24	119.5
C15—C14—C19	118.45 (14)	C25—C24—H24	119.5
C15—C14—C1	121.29 (13)	C10—C9—C8	121.41 (16)
C19—C14—C1	120.02 (13)	C10—C9—H9	119.3
N1—C1—C14	109.01 (10)	C8—C9—H9	119.3
N1—C1—C2	107.22 (10)	C18—C19—C14	120.63 (17)
C14—C1—C2	110.28 (11)	C18—C19—H19	119.7
N1—C1—C20	105.34 (10)	C14—C19—H19	119.7
C14—C1—C20	112.28 (10)	C5—C4—C3	120.32 (15)
C2—C1—C20	112.42 (10)	C5—C4—H4	119.8
C6—C7—C2	118.75 (13)	C3—C4—H4	119.8
C6—C7—C8	122.32 (13)	C11—C10—C9	119.86 (17)
C2—C7—C8	118.88 (12)	C11—C10—H10	120.1
C5—C6—C7	121.16 (14)	C9—C10—H10	120.1
C5—C6—H6	119.4	C10—C11—C12	120.78 (17)
C7—C6—H6	119.4	C10—C11—H11	119.6
C20—C25—C24	120.47 (14)	C12—C11—H11	119.6
C20—C25—H25	119.8	C17—C18—C19	120.67 (18)
C24—C25—H25	119.8	C17—C18—H18	119.7
C4—C3—C2	120.80 (14)	C19—C18—H18	119.7
C4—C3—H3	119.6	C17—C16—C15	120.05 (18)
C2—C3—H3	119.6	C17—C16—H16	120.0
C22—C21—C20	121.45 (14)	C15—C16—H16	120.0
C22—C21—H21	119.3	C18—C17—C16	119.70 (17)
C20—C21—H21	119.3	C18—C17—H17	120.2
C23—C22—C21	120.30 (15)	C16—C17—H17	120.2
C23—C22—H22	119.8		

### Hydrogen-bond geometry (Å, °)

**Table:**

*D*—H···*A*	*D*—H	H···*A*	*D*···*A*	*D*—H···*A*
C21—H21···O1	0.93	2.42	3.044 (2)	124
C24—H24···Cg1^i^	0.93	3.27	3.864 (3)	136
C6—H6···Cg2^ii^	0.93	3.16	3.932 (4)	142
C10—H10···Cg3^iii^	0.93	2.97	3.839 (4)	154

Symmetry codes: (i) −*x*, −*y*+1, −*z*; (ii) *x*, −*y*+3/2, *z*−1/2; (iii) −*x*, −*y*+2, −*z*.
